# The existence of an insulin-stimulated glucose and non-essential but not essential amino acid substrate interaction in diabetic pigs

**DOI:** 10.1186/1471-2091-12-25

**Published:** 2011-05-23

**Authors:** Sietse J Koopmans, Jan VanderMeulen, Jan Wijdenes, Henk Corbijn, Ruud Dekker

**Affiliations:** 1 BioMedical Research of Wageningen University and Research Center, Lelystad, The Netherlands; 2 Department of Animal Sciences, Adaptation Physiology Group of Wageningen University, Wageningen, The Netherlands; 3 Laboratory of Endocrinology and Metabolism of Wageningen University and Research Center, Lelystad, The Netherlands; 4 Experimental Animal Services of Wageningen University and Research Center, Lelystad, The Netherlands

## Abstract

**Background:**

The generation of energy from glucose is impaired in diabetes and can be compensated by other substrates like fatty acids (Randle cycle). Little information is available on amino acids (AA) as alternative energy-source in diabetes. To study the interaction between insulin-stimulated glucose and AA utilization in normal and diabetic subjects, intraportal hyperinsulinaemic euglycaemic euaminoacidaemic clamp studies were performed in normal (n = 8) and streptozotocin (120 mg/kg) induced diabetic (n = 7) pigs of ~40-45 kg.

**Results:**

Diabetic vs normal pigs showed basal hyperglycaemia (19.0 ± 2.0 vs 4.7 ± 0.1 mmol/L, *P *< .001) and at the level of individual AA, basal concentrations of valine and histidine were increased (*P *< .05) whereas tyrosine, alanine, asparagine, glutamine, glutamate, glycine and serine were decreased (*P *< .05). During the clamp, diabetic vs normal pigs showed reduced insulin-stimulated glucose clearance (4.4 ± 1.6 vs 16.0 ± 3.0 mL/kg·min, *P *< .001) but increased AA clearance (166 ± 22 vs 110 ± 13 mL/kg· min, *P *< .05) at matched arterial euglycaemia (5-7 mmol/L) and euaminoacidaemia (2.8-3.5 mmol/L). The increase in AA clearance was mainly caused by an increase in non-essential AA clearance (93.6 ± 13.8 vs 46.6 ± 5.4 mL/kg·min, *P *< .01), in particular alanine (14.2 ± 2.4 vs 3.2 ± 0.4 mL/kg·min, *P *< .001). Essential AA clearance was largely unchanged (72.9 ± 8.5 vs 63.3 ± 8.5 mL/kg· min), however clearances of threonine (*P *< .05) and tyrosine (*P *< .01) were increased in diabetic vs normal pigs (8.1 ± 1.3 vs 5.2 ± 0.5, and 14.3 ± 2.5 vs 6.4 ± 0.7 mL/kg· min, respectively).

**Conclusions:**

The ratio of insulin-stimulated glucose versus AA clearance was decreased 5.4-fold in diabetic pigs, which was caused by a 3.6-fold decrease in glucose clearance and a 2.0-fold increase in non-essential AA clearance. In parallel with the Randle concept (glucose - fatty acid cycle), the present data suggest the existence of a glucose and non-essential AA substrate interaction in diabetic pigs whereby reduced insulin-stimulated glucose clearance seems to be partly compensated by an increase in non-essential AA clearance whereas essential AA are preferentially spared from an increase in clearance.

## Background

The interaction between glucose and nonesterified fatty acids (NEFA) for oxidation in tissues or at the whole body level has been documented in many animal and human studies [1-3]. The glucose - NEFA cycle was first described by Randle and coworkers [4]. In essence they showed that the metabolic relationship between the substrates glucose and NEFA is reciprocal and not dependent. High plasma glucose concentrations promote glucose oxidation and inhibit NEFA oxidation. Vice versa, high plasma NEFA concentrations promote NEFA oxidation and inhibit glucose oxidation. Apart from the fact that mass action drives the glucose - NEFA cycle, substrate competition is also mediated by insulin. Insulin stimulates glucose oxidation and inhibits NEFA oxidation. The basic principles of the glucose - NEFA cycle also seem to apply for glucose and amino acids (AA) and therefore the existence of a glucose -AA cycle was proposed [5-8].

The purpose of substrate competition is to meet the energy demand of the body by oxidation of different substrates, i.e. glucose, NEFA or AA, depending on the availability of the substrates. At fasting, when plasma glucose, AA, and insulin levels are low, plasma NEFA levels will increase due to lipolysis from body fat, and NEFA will be primarily oxidized. After a meal, when plasma glucose, AA, and insulin levels are high, glucose and to a lesser extent AA will be oxidized.

In diabetic subjects however, the orchestrating role of substrate competition to channel energy oxidation depending on substrate availability is out of balance. The diabetic state is characterized by hyperglycaemia and insulin resistance and, as such, the metabolic handling of substrates like glucose, NEFA and AA is disturbed. For instance, diabetic subjects are hyperglycaemic but show impaired (insulin-stimulated) glucose oxidation and increased NEFA oxidation at elevated plasma NEFA concentrations [1,9,10]. An increased rate of NEFA oxidation may thus represent a mechanism to meet the energy demand of diabetic subjects. There is inconsistent information whether AA play a role in the unbalance of substrate competition in diabetic subjects [11]. Both increased [12,13] and unchanged [14-16] AA oxidation has been reported in studies with diabetic patients. The inconsistency may be caused by differences in study design: severity of diabetes, treatment with medication or with diets, used substrate methodology and control over plasma glucose and AA concentrations during the study, all play a role in the balance of substrate competition in diabetic subjects [11].

The aim of the present study was to characterize and compare insulin-stimulated glucose and AA utilization in normal and streptozotocin (STZ) diabetic pigs by means of the hyperinsulinaemic, euglycaemic, euaminoacidaemic clamp technique (additional file 1), thereby avoiding differences in substrate fluxes by mass action. The pig was chosen as animal model for the study of translational metabolism because of the many similarities between pigs and humans. Both species are of equal size and they are omnivores with similar food intake patterns, digestion and metabolism [17,18].

## Results

### Experiment 1

The insulin-stimulated individual AA clearance rates in 4 normal and 4 diabetic pigs, as calculated from the first clamp study when using the AA mixure from Table 1 are presented in Table 2. In diabetic pigs, the clearance rates of some essential AA like arginine, phenylalanine, threonine and tyrosine were increased 2-3 fold whereas the clearance rates of all non-essential AA were increased 2-4 fold.

**Table 1:** Tailor-made mixture of 20 amino acids (g/L) designed for infusion during the hyperinsulinaemic euglycaemic euaminoacidaemic clamp in normal pigs.

Arginine, 3.44	Methionine, 1.56	Valine, 3.68	Glutamine, 6.71
Histidine-HCl, 2.00	Phenylalanine, 2.44	Alanine, 4.30	Glutamate, 4.77

Isoleucine, 3.01	Threonine, 2.87	Asparagine, 1.70	Glycine, 7.26

Leucine, 5.39	Tryptophan, 0.90	Aspartate, 0.23	Proline, 4.56

Lysine-HCl 3.59	Tyrosine, 2.29	Cysteine, 0.58	Serine, 3.50

Total amino acid concentration is 65 g/L. Essential amino acids are ARG through VAL. Non-essential amino acids are ALA through SER.

**Table 2:** Insulin-stimulated amino acid clearance rates during the steady state phase of the hyperinsulinaemic euglycaemic euaminoacidaemic clamp in 4 normal and 4 diabetic pigs in experiment 1, when both normal and diabetic pigs were infused with an amino acid mixture (Table 1) designed for normal pigs.

Plasma amino acid	Normal pigs (mL/kg·min )	Diabetic pigs (mL/kg·min)
Essential:		
Arginine	4.3 ± 0.8	10.4 ± 2.3*
Histidine	4.0 ± 0.8	4.2 ± 0.5
Isoleucine	5.2 ± 0.9	5.5 ± 1.6
Leucine	5.9 ± 0.9	6.8 ± 1.2
Lysine	5.6 ± 1.2	6.9 ± 0.5
Methionine	9.2 ± 1.1	16.3 ± 2.0
Phenylalanine	5.8 ± 0.8	10.2 ± 1.6*
Threonine	4.5 ± 0.4	8.9 ± 0.8^‡^
Tryptophan	2.1 ± 0.3	4.3 ± 1.2
Tyrosine	5.6 ± 0.6	19.8 ± 5.5*
Valine	2.3 ± 0.3	2.5 ± 0.7
		
Non-essential:		
Alanine	2.8 ± 0.2	10.7 ± 2.8*
Asparagine	6.5 ± 0.6	15.0 ± 1.9^†^
Aspartate	1.7 ± 0.2	4.8 ± 1.1*
Cysteine	2.4 ± 0.3	5.8 ± 1.1*
Glutamine	1.7 ± 0.1	5.1 ± 1.3*
Glutamate	4.1 ± 0.4	14.3 ± 2.1^†^
Glycine	2.3 ± 0.3	6.6 ± 1.6*
Proline	3.9 ± 0.4	6.9 ± 0.9*
Serine	5.3 ± 0.6	14.2 ± 3.1*

Mean ± SEM, * *P *< .05, † *P *< .01 and ‡ *P *< .001 compared to normal pigs.

During the clamp studies in diabetic pigs the steady state plasma AA concentrations were compared to the corresponding preclamp (basal) AA concentrations (data not shown) in order to adjust the AA mixture to the diabetic status. After each of the 3 clamp studies in diabetic pigs, the AA mixture was further finetuned to optimally meet the utilization rates per individual AA in diabetic pigs. The final AA mixture for diabetic pigs is presented in Table 3.

**Table 3:** Tailor-made mixture of 20 amino acids (g/L) designed for infusion during the hyperinsulinaemic euglycaemic euaminoacidaemic clamp in diabetic pigs.

Arginine, 2.78	Methionine, 1.85	Valine, 3.39	Glutamine, 3.29
Histidine-HCl, 2.47	Phenylalanine, 2.34	Alanine, 6.46	Glutamate, 4.32

Isoleucine, 3.28	Threonine, 2.85	Asparagine, 2.39	Glycine, 5.31

Leucine, 6.03	Tryptophan, 0.53	Aspartate, 0	Proline, 6.20

Lysine-HCl 5.42	Tyrosine, 2.85	Cysteine, 0.77	Serine, 2.49

Total amino acid concentration is 65 g/L. Essential amino acids are ARG through VAL. Non-essential amino acids are ALA through SER.

### Experiment 2

**Figure 1:**
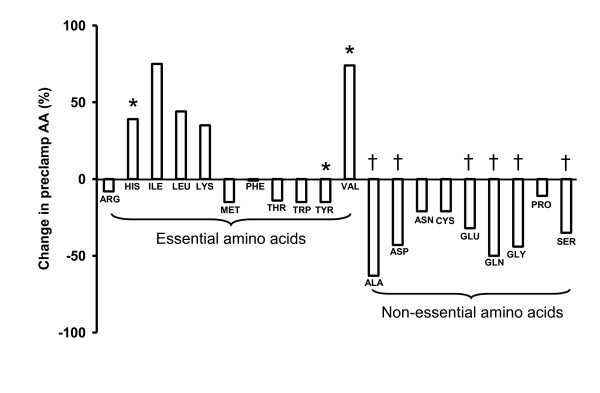
**The diabetes-induced change (%) in preclamp (basal) plasma amino acid concentrations when comparing diabetic pigs (Table 5, n = 7) with normal pigs (Table 4, n = 8)**. * *P *< .05 and † *P *< .001.

Diabetic pigs (n = 7) versus normal pigs (n = 8) showed basal hyperglycaemia (Tables 4 and 5, *P *< .001), reduced plasma concentrations of total AA and increased plasma concentrations of urea (Tables 4 and 5, both *P *< .05) and at the level of individual AA (Figure 1), some essential AA concentrations were increased (valine and histidine, *P *< .05) whereas one essential AA (tyrosine, *P *< .05) and the non-essential AA alanine, asparagine, glutamine, glutamate, glycine and serine were decreased (*P *< .05).

**Table 4:** Plasma insulin, glucose, lactate, total amino acids and urea concentrations are shown at preclamp (basal) and at steady state during the hyperinsulinaemic euglycaemic euaminoacidaemic clamp in 8 normal pigs infused with an AA-mixture (Table 1) designed for normal pigs.

Plasma concentration	Preclamp (basal)	Steady state clamp
Insulin (mU/L)	7 ± 3	25 ± 4
Glucose (mmol/L)	4.7 ± 0.1	5.0 ± 0.2
Lactate (mmol/L)	0.7 ± 0.1	0.8 ± 0.1
Total amino acids (mmol/L)	3.5 ± 0.1	3.5 ± 0.1
Urea (mmol/L)	2.9 ± 0.2	2.6 ± 0.2

Means ± SEM

**Table 5:** Plasma insulin, glucose, lacate, total amino acids and urea concentrations are shown at preclamp (basal) and at steady state during the hyperinsulinaemic euglycaemic euaminoacidaemic clamp in 7 diabetic pigs infused with an AA-mixture (Table 3) designed for diabetic pigs.

Plasma concentration	Preclamp (basal)	Steady state clamp
Insulin (mU/L)	5 ± 4	26 ± 6
Glucose (mmol/L)	19.0 ± 2.0	6.8 ± 0.7
Lactate (mmol/L)	0.7 ± 0.1	1.2 ± 0.4
Total amino acids (mmol/L)	2.9 ± 0.1	2.8 ± 0.1
Urea (mmol/L)	5.1 ± 0.5	4.7 ± 0.5

Means ± SEM

In normal pigs, at steady state during the clamp study with the tailor made AA mixture for normal pigs (Table 1), plasma insulin concentrations were increased approximately 4-fold (*P *< .001) over preclamp concentrations whereas the concentrations of glucose, lactate, total AA, urea and all individual AA (less than 15%) did not change significantly (Tables 4 and 6). The coefficients of variation of individual AA concentrations were less than 21% in the preclamp situation and less than 12% in the steady state situation.

**Table 6:** Individual amino acid concentrations are shown at preclamp (basal) and at steady state during the hyperinsulinaemic euglycaemic euaminoacidaemic clamp in 8 normal pigs infused with an AA-mixture (Table 1) designed for normal pigs.

Plasma amino acid	Preclamp (basal) (μ mol/L)		Steady state clamp (μ mol/L)		Deviation from basal	
		VC (%)		VC (%)	(%)	*P*-value
Essential:						
Arginine	109 ± 4	8	100 ± 4	7	-8	
Histidine	46 ± 3	11	50 ± 3	6	9	
Isoleucine	111 ± 12	5	94 ± 10	6	-15	
Leucine	168 ± 7	4	160 ± 12	5	-5	
Lysine	85 ± 6	12	72 ± 6	11	-15	
Methionine	27 ± 1	7	25 ± 1	7	-7	
Phenylalanine	49 ± 2	8	50 ± 3	5	2	
Threonine	121 ± 10	8	121 ± 7	6	0	
Tryptophan	39 ± 3	13	43 ± 2	4	10	
Tyrosine	56 ± 5	7	53 ± 2	6	-5	
Valine	291 ± 28	6	259 ± 13	5	-11	
						
Non-essential:						
Alanine	422 ± 28	8	418 ± 34	7	-1	
Asparagine	49 ± 4	11	46 ± 3	7	-6	
Aspartate	20 ± 2	21	18 ± 1	12	-10	
Cysteine	35 ± 4	6	35 ± 3	4	0	
Glutamine	551 ± 27	6	585 ± 22	5	6	
Glutamate	182 ± 10	9	178 ± 10	10	-2	
Glycine	799 ± 32	6	866 ± 33	4	8	
Proline	201 ± 12	4	214 ± 7	4	6	
Serine	142 ± 6	13	129 ± 8	7	-9	

Means ± SEM, VC = coefficient of variation.

In diabetic pigs, at steady state during the clamp study with the tailor made AA mixture for diabetic pigs (Table 3), plasma insulin concentrations were increased approximately 5-fold (*P *< .001) over preclamp concentrations, plasma glucose concentrations were reduced to the euglycaemic range and the concentrations of lactate, total AA, urea and all individual AA did not change significantly (Tables 5 and 7). The coefficients of variation of individual AA concentrations were less than 15% in the preclamp situation and less than 11% in the steady state situation.

**Table 7:** Individual amino acid concentrations are shown at preclamp (basal) and at steady state during the hyperinsulinaemic euglycaemic euaminoacidaemic clamp in 7 diabetic pigs infused with an AA-mixture (Table 3) designed for diabetic pigs.

Plasma amino acid	Preclamp (basal) (μ mol/L)		Steady state clamp (μ mol/L)		Deviation From basal	
		VC (%)		VC (%)	(%)	*P*-value
Essential:						
Arginine	99 ± 11	6	83 ± 9	8	-16	
Histidine	64 ± 6	3	69 ± 5	6	8	
Isoleucine	194 ± 39	4	144 ± 25	6	-26	
Leucine	242 ± 41	5	214 ± 21	6	-12	
Lysine	114 ± 13	8	146 ± 13	6	28	
Methionine	23 ± 2	8	27 ± 2	10	17	
Phenylalanine	48 ± 4	6	50 ± 3	5	4	
Threonine	105 ± 13	6	109 ± 12	7	4	
Tryptophan	33 ± 2	7	32 ± 3	7	-3	
Tyrosine	39 ± 2	9	39 ± 3	8	0	
Valine	507 ± 79	3	363 ± 51	5	-28	
						
Non-essential:						
Alanine	158 ± 32	6	180 ± 16	7	14	
Asparagine	28 ± 4	15	34 ± 5	10	21	
Aspartate	16 ± 3	6	19 ± 5	7	19	
Cysteine	28 ± 3	4	34 ± 3	6	21	0.09
Glutamine	375 ± 28	2	357 ± 33	5	-5	
Glutamate	90 ± 9	7	96 ± 8	11	7	
Glycine	444 ± 72	3	546 ± 57	6	23	
Proline	179 ± 13	6	211 ± 12	6	18	0.09
Serine	93 ± 7	8	91 ± 7	8	-2	

Means ± SEM. VC = coefficient of variation.

**Figure 2:**
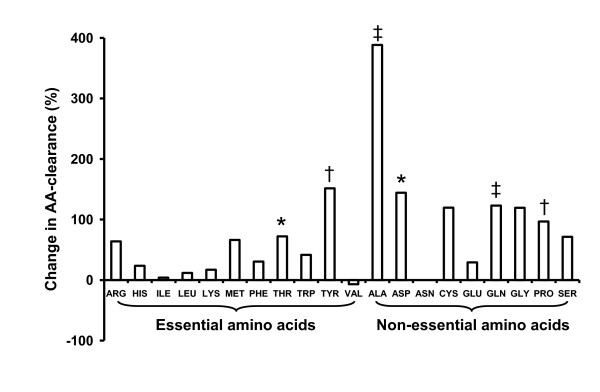
**The diabetes-induced change (%) in insulin-stimulated individual AA clearance rates when comparing diabetic pigs (Table 6, n = 7) with normal pigs (Table 6, n = 8)**. * *P *< .05, † *P *< .01 and ‡ *P *< .001.

**Figure 3:**
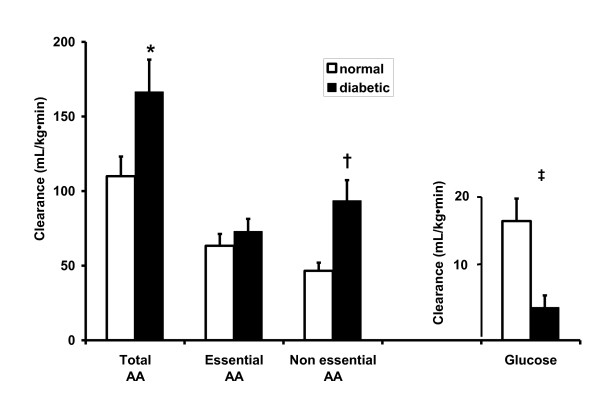
**The insulin-stimulated total amino acid (AA), essential AA, non-essential AA and glucose clearance rates in normal (n = 8) and diabetic pigs (n = 7)**. * *P *< .05, † *P *< .01 and ‡ *P *< .001.

The insulin-stimulated individual AA clearance rates in normal and diabetic pigs, are presented in Table 8. In diabetic pigs, the clearance rates of the essential AA threonine and tyrosine were increased approximately 2-fold and the clearance rates of the non-essential AA alanine, asparagine, glutamate and proline were increased 2-4 fold. The corresponding %-change in individual AA clearance rates from the normal to the diabetic status is depicted in Figure 2. When the AA clearance rates are subdivided in total AA, essential AA and non-essential AA (Figure 3), the clearance rates of total AA (166 ± 22 vs 110 ± 13 mL/kg·min, *P *< .05) and non-essential AA (93.6 ± 13.8 vs 46.6 ± 5.4 mL/kg·min (*P *< .01) were increased in diabetic vs normal pigs. Essential AA clearance was largely unchanged (72.9 ± 8.5 vs 63.3 ± 8.5 mL/kg·min). Glucose clearance rate (Figure 3) was reduced approximately 4-fold in diabetic vs normal pigs (4.4 ± 1.6 vs 16.0 ± 3.0 mL/kg·min, *P *< .001). Taken together, the ratio of insulin-stimulated glucose versus AA clearance was decreased 5.4-fold in diabetic pigs, which was caused by a 3.6-fold decrease in glucose clearance and a 2.0-fold increase in non-essential AA clearance.

**Table 8:** Insulin-stimulated amino acid clearance rates during the steady state phase of the hyperinsulinaemic euglycaemic euaminoacidaemic clamp in 8 normal and 7 diabetic pigs.

Plasma amino acid	Normal pigs (mL/kg·min)	Diabetic pigs (mL/kg·min)
Essential:		
Arginine	5.2 ± 0.5	7.2 ± 1.4
Histidine	5.3 ± 0.7	5.7 ± 0.5
Isoleucine	7.0 ± 1.3	6.2 ± 0.7
Leucine	7.3 ± 1.0	7.3 ± 0.7
Lysine	7.4 ± 0.9	7.2 ± 1.1
Methionine	11.4 ± 1.4	16.1 ± 2.4
Phenylalanine	8.2 ± 1.3	9.3 ± 1.1
Threonine	5.2 ± 0.5	8.1 ± 1.3*
Tryptophan	2.7 ± 0.3	3.0 ± 0.6
Tyrosine	6.4 ± 0.7	14.3 ± 2.5^†^
Valine	3.6 ± 0.8	2.8 ± 0.4
		
Non-essential:		
Alanine	3.2 ± 0.4	14.2 ± 2.4^‡^
Asparagine	9.5 ± 1.8	22.1 ± 5.4*
Aspartate	2.0 ± 0.2	0 ± 0
Cysteine	4.1 ± 0.8	6.7 ± 1.2
Glutamine	2.1 ± 0.2	2.4 ± 0.6
Glutamate	5.0 ± 0.6	10.6 ± 1.3^‡^
Glycine	2.9 ± 0.3	5.2 ± 1.5
Proline	4.9 ± 0.5	8.6 ± 0.9^†^
Serine	6.5 ± 0.7	9.5 ± 1.9

Normal pigs were infused with an amino acid mixture (Table 1) designed for normal pigs and diabetic pigs were infused with an amino acid mixture (Table 3) designed for diabetic pigs.

Mean ± SEM, * *P *< .05, † *P *< .01 and ‡ *P *< .001 compared to normal pigs.

## Discussion

This study demonstrates that in diabetic pigs insulin resistance for glucose utilization is accompanied by insulin hypersensitivity for non-essential AA utilization. In parallel with the Randle concept (glucose - NEFA cycle), the present data suggest the existence of a glucose - non-essential AA substrate interaction in diabetic pigs whereby reduced insulin-stimulated glucose clearance seems to be compensated by increased non-essential AA clearance.

Insulin resistance for glucose metabolism in diabetic subjects has been argued to be a primary metabolic defect caused by glucose and lipid toxicity [1,17] and insulin hypersensitivity for non-essential AA clearance, as shown in the present study, seems therefore a secondary response, probably a compensatory mechanism to warrant sufficient energy supply to body tissues of diabetic subjects.

It has previously been shown that substrate competition, apart from glucose - NEFA, can take place between glucose and AA. Infusion of an AA mixture reduced insulin-stimulated glucose disposal, and more specifically glucose oxidation, in healthy man [2,5,8]. Theoretically the reduction in glucose oxidation was caused by increased AA oxidation. In our study with diabetic pigs, the low glucose utilization rate coincided with a high utilization rate of non-essential AA. Plasma urea concentrations remained high during the clamp studies in diabetic pigs which suggests that AA oxidation was not inhibited by insulin. Therefore, the increase in non-essential AA clearance in diabetic pigs most likely reflects increased AA oxidation.

### Glucose and amino acid utilization in normal and diabetic pigs

In the present study, nutrient utilization was expressed as nutrient clearance in mL/kg·min. Another approach is to express nutrient utilization as mg/kg·min. This is valid for glucose and total AA because nutrient utilization rates were determined at matched plasma glucose and AA concentrations (euglycaemia and near euaminoacidaemia), and therefore the masses of glucose and AA which were utilized by the body can be directly compared between normal and diabetic pigs. The insulin-stimulated net utilization of glucose (14.2 ± 2.3 mg/kg· min, i.e. ~920 g/day) was 8.4-times greater than the net utilization of AA (1.7 ± 0.2 mg/kg· min, i.e. ~110 g/day) in normal pigs. This is largely in accordance with our previous observations [19] where we found a ratio of ~10:1 in the net utilization of glucose compared to AA in normal pigs. In diabetic pigs however, the insulin-stimulated net utilization of glucose (4.6 ± 1.6 mg/kg· min, i.e. ~265 g/day) was almost equal to the net utilization of AA (3.4 ± 1.1 mg/kg· min, i.e. ~196 g/day). When transforming the net utilization rates to generation of gross energy from nutrients, the following calculation can be made: The combustion energy (KJ/g substrate) and net ATP production (mol/g substrate) for glucose vs AA vs NEFA are 15.6 and 0.211 vs 22.6 and 0.218 vs 38.2 and 0.504, respectively [20]. This implies that the 2-fold increase (from 1.7 to 3.4 mg/kg·min ) in AA utilization in diabetic pigs does not fully compensate for the 3.1-fold decrease (from 14.2 to 4.6 mg/kg·min ) in glucose utilization in terms of energy transfer. Energy content of glucose and AA is similar and therefore it can be concluded that the remainder of energy compensation in diabetic pigs must come from increased NEFA utilization to fill the existing energy gap (3.1 divided by 2 = 1.55) between a 3.1-fold decrease in glucose utilization and a 2-fold increase in AA utilization. Energy content of NEFA is approximately 2-fold higher compared to glucose and therefore it can be calculated that NEFA utilization must have been increased 1.275-fold (1.55 divided by 2) under the present experimental conditions in diabetic pigs.

### Essential and non-essential amino acid utilization in diabetic pigs

Adult pigs require a core of nine AA for maintenance and productive purposes which are called "essential". In young growing pigs, like the pigs used in the present study, two additional AA are considered (conditionally) essential: arginine and tyrosine. Other amino acids which pigs are able to synthesize are termed "non-essential" [21].

With respect to non-essential AA, alanine is the major AA utilized by the liver as a substrate for gluconeogenesis [22]. The plasma concentration of alanine reflects the algebraic sum of its release from peripheral tissue and rates of utilization by the liver for gluconeogenesis. Plasma alanine concentrations were low in diabetic pigs indicating that release of alanine is lower than its utilization. Our data show that insulin-stimulated alanine utilization was high in diabetic pigs, which fits with the general observation that gluconeogenesis from alanine is increased in diabetes [23].

Essential AA were preferentially spared from increased utilization in diabetic pigs, as shown for phenylalanine and leucine in previous studies in diabetic humans [14-16,24]. This may represent a protective mechanism to maintain body protein integrity. However, the utilization rates of two essential AA, threonine and tyrosine, were increased in diabetic pigs. Threonine, when catabolised, is dehydrated first to alpha-ketobutyrate, which is then converted to propionyl CoA, the precursor of succinyl CoA. Through threonine metabolism pyruvate is formed, which enters the Krebs Cycle [25]. Therefore threonine may compensate for the reduction in glucose-related energy in diabetes. However, threonine is also involved in many physiological functions, in particular those of immune system functionality [20,26], gut mucosal repair processes [21] and threonine deficiency inhibits growth and reduces body water content [21], thereby possibly worsening dehydration in diabetes. Tyrosine is the immediate precursor for production of the neurotransmitters and hormones dopamine, adrenaline, noradrenaline, thyroxine and the antioxidant melanin, and as such involved in the regulation of metabolism and inhibition of inflammation [20,26]. Abnormalities in metabolism and inflammation are characteristic features of diabetes [27]. A deficiency in some essential AA like threonine and tyrosine may therefore contribute to the development of secondary complications and the health problems of diabetic patients, i.e. increased susceptibility to infections and reduced nervous system functionality.

### Infused amino acid mixtures

It can be argued that the chosen composition of the infused AA mixture played a major role in the balance between glucose and AA utilization. There are two arguments against this assumption. First, the composition of the AA mixture was determined by the endogenous AA fluxes in the pig. Basal plasma AA concentrations were kept constant at steady state of the clamp and this approach determined the composition of the AA mixture. Second, two different AA mixtures were used in the present study. One AA mixture (Table 1) contained 48% essential and 52% non-essential AA, the other AA mixture (Table 3) contained 52% essential and 48% non-essential AA. Both AA mixtures yielded comparable results with respect to the increase in clearance rates of non-essential AA in diabetic pigs. Minor differences were observed at the level of some individual AA like arginine, phenylalanine, cysteine, glutamine, glycine and serine. Therefore the conclusion with respect to the existence of a glucose - non-essential AA substrate cycle in diabetic pigs is valid for both AA mixtures, i.e. a mixture which fits normal pigs (experiment 1) and a mixture which fits diabetic pigs (experiment 2).

### Limitations of the study

Data were collected by means of the hyperinsulinaemic euglycaemic euaminoacidaemic clamp technique which reflects the nutrient uptake capacity of the body. Linking these data to the Randle cycle is a debatable issue because oxidation of nutrients was not measured in the present study. Referring to the plasma urea concentrations as a crude measure of AA oxidation is only partly valid. During the clamp, nutrients disappear from the blood into tissues and the intracellular fate of these nutrients is unknown. AA could be used for protein synthesis or oxidation. Young pigs have a high capacity for growth and therefore it is expected that a large portion of the infused AA is used for protein accretion and not for oxidation. It is uncertain whether the capacity for protein accretion is unaltered during hyperinsulinaemic euglycaemic euaminoacidaemic conditions in diabetic pigs. However, there are some indications that this is the case: 1) Lysine is the first limiting AA for protein accretion and body weight gain in pigs [28]. In the present study, lysine clearance was not different in diabetic and control pigs. 2) Diabetes seems not to be associated with insulin resistance for protein synthesis and protein degradation [29,30]. 3) At identical dietary energy intake, average weekly growth of control pigs was ~4 kg whereas growth of diabetic pigs was ~1 kg with an urinary glucose excretion of ~2 kg. This is in line with our previous observations [17]. This suggests that dietary energy efficacy is reduced ~4-fold in hyperglycaemic diabetic pigs which seems to be mainly caused by loss of energy via the urine. Under the present euglycaemic clamp conditions however, when urinary glucose loss is negligible, the capacity for growth seems similar in control and diabetic pigs.

Taken together, the interpretation of the data with respect to the Randle cycle should be read with the reservation in mind that oxidation was not measured in the present study.

## Conclusions

Studies so far have established that excess exogenous AA infusion competes with glucose for uptake by insulin-sensitive tissues in healthy individuals [2,5,8]. Whether this substrate competition is in effect in diabetic subjects was the research focus of the present study. We conclude that a glucose - AA cycle exists in diabetic subjects but the nature of substrate competition seems different from healthy subjects. Given the fact that glucose uptake is suppressed and considered to be the primary metabolic defect in diabetic subjects, the increase in non-essential AA uptake seems a compensatory mechanism to provide the body with sufficient energy. Therefore the glucose - AA cycle in diabetic subjects may be designated as "compensatory" in stead of "competitive" with regard to the substrate interaction, being a glucose - non essential AA substrate compensation.

## Methods

The principles of laboratory animal care (NIH publication no. 85-23, revised 1985) were followed. Experimental protocols describing the management, surgical procedures, and animal care were reviewed and approved by the ASG-Lelystad Animal Care and Use Committee (Lelystad, The Netherlands).

### Animals, housing, diets and surgery

Twenty-four crossbred barrows (Dutch Landrace × Yorkshire × Finnish Landrace) with an initial body weight of 30-35 kg were used in this study. Two weeks before surgery the pigs were kept in specially designed metabolic pens (1.15 × 1.35 m) and adapted to the light/dark cycle (lights on at 0500 h and off at 1900 h) and the feeding schedule. The pigs were fed a commercial pig diet (5% crude fat, 16% crude protein, 41% starch and sugars, 20% non-starch polysaccharides, 6% ash and 12% water; Startbrok; Agrifirm, Meppel, The Netherlands). The pigs were fed twice daily (at 0600 h and 1600 h) at a feeding level of 2.8 times maintenance requirements for metabolizable energy (ME). This corresponded with a feeding level of 1109 kJ ME/kg BW^0.75 ^(metabolic weight of the pig) which is close to the ad libitum feeding level for pigs [28]. Water was always available ad libitum.

Pigs were provided with 3 permanent blood vessel catheters in the jugular vein, carotid artery and portal vein, as previously described [19,31]. During the period between surgery and the clamp study (two weeks post-surgery), the pigs were habituated to the blood sampling procedure. The carotid artery was used for blood sampling and the jugular vein catheter was used as a back-up in case of a malfunctioning arterial catheter. The portal vein catheter was used for the infusion of fluids during the hyperinsulinaemic euglycaemic euaminoacidaemic clamp experiments. During the blood sampling procedure, the catheters were flushed with physiological saline and sealed off with physiological saline containing 5 IU heparin per mL. To avoid activation of lipoprotein lipase by heparin, care was taken that minimal amounts of heparin entered the blood stream and from experience we know that this sampling procedure does not affect plasma NEFA concentrations.

### Normal and diabetic pigs

After surgery, half of the pigs were treated with streptozotocin (STZ; Pharmacia & Upjohn Company, Kalamazoo, MI., 120 mg/kg) as described previously [17]. Two weeks thereafter, 1 pig showed fasting plasma glucose concentrations <10 mmol/L and was excluded from the study. Other diabetic pigs remained non-ketotic throughout the study as determined by analysis of acetoacetic acid in urine.

### Hyperinsulinaemic euglycaemic euaminoacidaemic clamp technique

The intraportal hyperinsulinaemic euglycaemic euaminoacidaemic clamp technique was used to quantitate insulin-stimulated net utilization of plasma glucose and of individual AA in pigs [19,31-33]. Nutrient utilization was expressed as nutrient clearance (infusion rate of nutrients (in mol/kg·min ) divided by the plasma nutrient concentration (in mol/mL). When comparing blood nutrient fluxes at different nutrient concentrations in blood (in particular for some of the 20 AA), the calculation of nutrient clearance normalizes the nutrient fluxes for differences in plasma nutrient concentrations, thereby eliminating the mass action effect of concentration on flux and thus exposing whole body nutrient utilization at identical blood nutrient concentrations.

By portal infusion the physiological route of appearance of insulin (secreted from the pancreatic beta-cells), glucose and AA (from the intestine after food intake) is mimicked. Concentrations of insulin, glucose and AA are high in the portal vein during the intraportal clamp studies, and therefore the liver will be exposed to physiologically correct concentrations of insulin, glucose and AA. This allows the liver to fully exert its metabolic control function in the body. For instance, most glucogenic AA (mainly alanine) are metabolized by the liver whereas branched-chain AA (valine, (iso)leucine) are metabolized in peripheral tissues. Considering the foregoing argumentation, it is important to impose a correct portal - peripheral gradient for insulin, glucose and all individual AA, resulting in a proper simulation of whole body insulin-stimulated rates of glucose and AA.

Insulin (Actrapid MC, porcine monocomponent, Novo, Copenhagen, Denmark), D-glucose (Merck, Darmstadt, Germany) and a tailor-made mixture of 20 AA (Sigma, Zwijndrecht, The Netherlands) (Tables 1 and 3) were prepared as sterile solutions and passed through a 0.22 μ m Millipore filter into sterile containers before use. Insulin was diluted in a saline solution containing 3% pig plasma in order to avoid sticking of insulin to the plastic containers and tubings. D-glucose and the AA were dissolved in water.

In the basal state, at 0600 h and 0615 h, two heparinized blood (10 mL) samples were collected. At 0630 h, hyperinsulinaemic euglycaemic euaminoacidaemic clamp experiments were started by a prime (26 mU/kg)-continuous (1.5 mU/kg·min) intraportal infusion of insulin for 6 hours via the portal catheter. Due to the insulin infusion, plasma glucose and AA concentrations declined and a variable intraportal infusion of a 33% D-glucose solution (330 g/L) and an AA solution (65 g/L) (Tables 1 and 3) were started and the infusion rates were adjusted every 10 (glucose solution) and 20 minutes (AA solution) to maintain the plasma glucose and phenylalanine concentrations at euglycaemic (5-7 mmol/L) and euaminoacidaemic (2.8-3.5 mmol/L) levels. Steady state conditions for plasma glucose and phenylalanine concentrations and the infusion rates of glucose and AA were achieved within 5 hours after initiation of the hyperinsulinaemic clamp and steady state calculations for whole body glucose and AA utilization were carried out during the last 40 minutes of the clamp (t = 320, 340 and 360 minutes). At 320, 340 and 360 minutes, blood (10 mL) samples were collected in heparinized tubes for measurement of plasma insulin, glucose, lactate, 20 individual AA, and urea concentrations.

### Experiments

In total, 12 non-diabetic (normal) and 11 diabetic pigs were studied with the hyperinsulinaemic euglycaemic euaminoacidaemic clamp technique in 2 experiments.

In experiment 1, normal (n = 4) and diabetic pigs (n = 4) were infused with insulin, glucose and an AA mixture specifically designed for normal pigs (Table 1). The infused, tailor made, AA mixture (Table 1) contained 20 AA in concentrations which were in proportion to the utilization rates per individual AA in the normal pig. By experience, this AA mixture was defined in previous experiments [19,31,32] allowing us to study insulin sensitivity in the absence of hypo- or hyperaminoacidaemia for individual AA. After data collection and calculation of the insulin-stimulated AA clearance rates in normal and diabetic pigs, the AA mixture was adjusted to diabetic pigs (for the use in the second experiment) in the following way:

To further fine-tune and adjust the AA mixture to the diabetic status the diabetic pigs were re-used for clamp studies twice more (after 2 week intervals). This led to a near-optimal AA mixture (Table 3) which contained 20 AA in concentrations which are in proportion to the utilization rates of individual AA in the diabetic pig.

In experiment 2, normal pigs (n = 8) were infused with insulin, glucose and an AA mixture specifically designed for normal pigs (Table 1) and the diabetic pigs (n = 7) were infused with insulin, glucose and an AA mixture specifically designed for diabetic pigs (Table 3), as calculated in experiment 1.

### Plasma and urine analyses

Blood samples collected in heparinized (150 USP. U. Lithium Heparin) or EDTA (ethylenediaminetetraacetic acid, 0.47 mol/L) tubes (10 mL Venoject, Terumo, Leuven, Belgium) were immediately chilled at 0°C on water with ice, and centrifuged at 4°C for 10 minutes at 3000 rpm. Plasma aliquots were stored at -80°C for later analyses.

Plasma insulin concentration was measured using a Delfia assay (test kit by Perkin Elmer Life Sciences Trust by Wallac Oy, Turku, Finland). This specific pig insulin assay was validated using pig insulin standards, as indicated before [17,19,31,32]. Plasma glucose and lactate concentrations were analyzed with a blood autoanalyzer of Radiometer (ABL and AML, Copenhagen, Denmark). Plasma urea concentration was analyzed by the method described by Gutmann & Bergmeyer [34].

Plasma tryptophan and phenylalanine concentrations were measured by reversed-phase liquid chromatography (HPLC System Gold, Beckman, Fullerton, CA, USA) using a C18 (Hypersil) column (Alltech, Deerfield, IL, USA), and detected with a fluorescence detector at 217 nm [35]. For rapid plasma phenylalanine determination during the clamp studies, blood samples (0.5 mL) were immediately centrifuged in a microcentrifuge for 0.5 min, 0.1 mL of a 8% salicylic acid (SSA) solution was added to 0.1 mL plasma, mixed thoroughly, centrifuged in a microcentrifuge for 0.5 minute and 0.02 mL of supernatant was injected in the reversed-phase HPLC system. Retention time for phenylalanine and tryptophan analyses were 4 and 6 minutes respectively. The concentrations of AA in plasma (except for tryptophan) were analyzed as described previously [35].

Ketones (acetoacetic acid) were determined in fresh urine by a reagent strip test (Ketostix, Bayer Diagnostics, Mijdrecht, The Netherlands).

### Statistical analyses

Each pig was an experimental unit. Results are expressed as means ± SEM and the criterion of statistical significance was set at *P *< .05. The data were subjected to the unpaired student's t-test of Genstat 5 [36] for determination of differences between two groups, respectively.

## List of abbreviations

AA = amino acids, ALA = alanine, ARG = arginine, ASP = asparagine, ASN = aspartate, BW = body weight, CYS = cysteine, GLU = glutamine, GLN = glutamate, GLY = glycine, HIS = histidine, ILE = isoleucine, LEU = leucine, LYS = lysine, ME = metabolizable energy, MET = methionine, NEFA = nonesterified fatty acids, PHE = phenylalanine, PRO = proline, SER = serine, STZ = streptozotocin, THR = threonine, TRP = tryptophan, TYR = tyrosine, VAL = valine.

## Authors' contributions

SJK was the principal investigator, involved in designing the study and writing the manuscript. JM was involved in developing the surgical techniques and writing the manuscript. JW was involved in developing and performing the amino acid analyses techniques. HC was involved in developing and performing the clamp technique in pigs. RD coordinated the study and performed statistical analyses. All authors participated in writing the final version of the manuscript.

## Supplementary Material

Additional file 1 **The hyperinsulinaemic euglycaemic euaminoacidaemic clamp in a pig**. A photograph showing blood sampling in a conscious pig. Click here for file
