# Supplementary figures and images for: The existence of an insulin-stimulated glucose and non-essential but not essential amino acid substrate interaction in diabetic pigs
Source: BMC Biochem. 2011 May 23;12:25. doi: 10.1186/1471-2091-12-25 (PMC3129298; doi:10.1186/1471-2091-12-25)

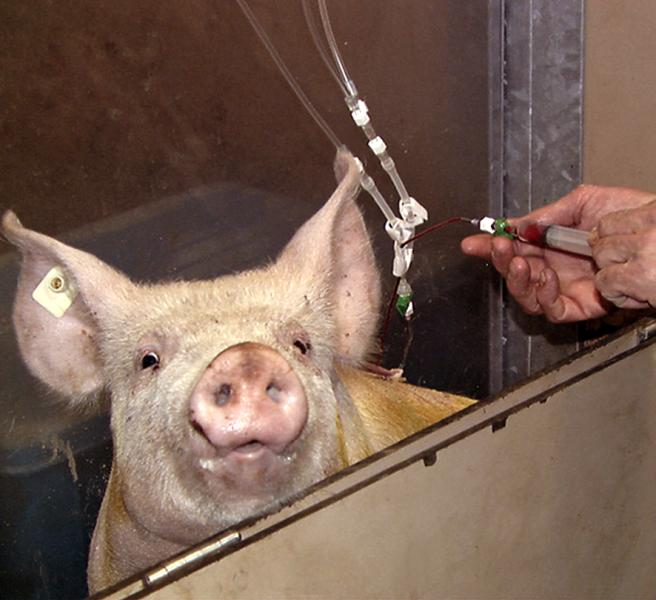

Supplement: Additional file 1 — The hyperinsulinaemic euglycaemic euaminoacidaemic clamp in a pig. A photograph showing blood sampling in a conscious pig. [file 1471-2091-12-25-S1.DOC]
